# Supplementary material for: Highly conserved shifts in ubiquitin-proteasome system (UPS) activity drive mitochondrial remodeling during quiescence
Source: Nat Commun. 2022 Aug 1;13:4462. doi: 10.1038/s41467-022-32206-2 (PMC9343427; doi:10.1038/s41467-022-32206-2)
Supplement: Supplementary file 1 — Supplementary Information [file 41467_2022_32206_MOESM1_ESM.pdf]

Supplementary Figure 1

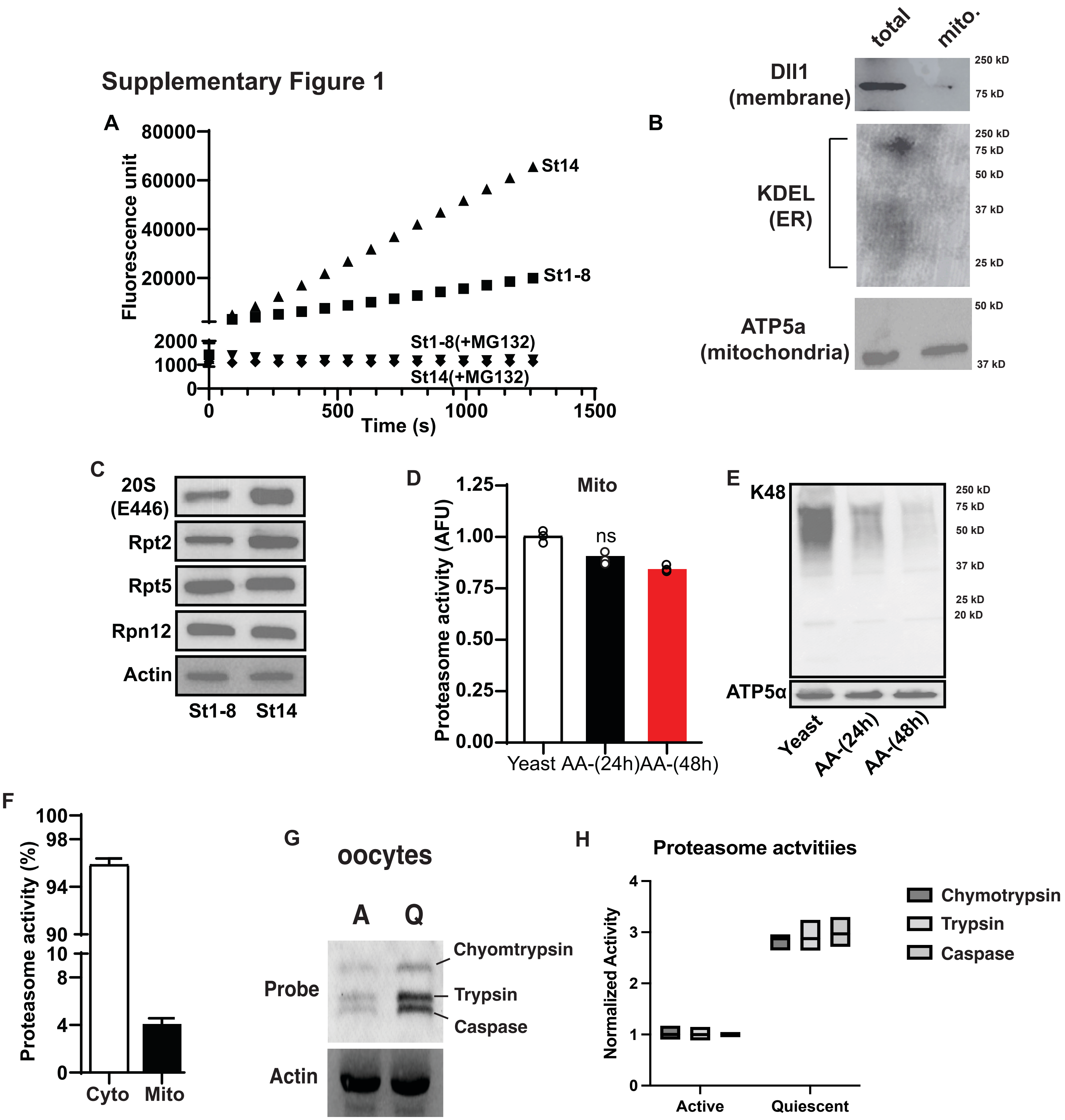

Supplementary Figure 1

A: Representative proteasome activity curve and control (+MG132) for total cell lysis in st1-8 and st14 eggs.

B: Western blots examining plasma membrane and ER levels in mitochondrial fractions. DLL1 and ATP5A blots show a signal bands where as KDEL show multiple bands consistent with detecting the ER localization signal of multiple proteins(n=3 independent experiments).

C: Denaturing Western blots to examine 20S core and 19S regulatory components of the proteasome in total cell lysis from st1-8 and st14 eggs(n=3 independent experiments).

D and E: Mitochondria-associated proteasome activity and K48 ubiquitin levels in st14 eggs from animals fed either a yeast or amino acid-deficient diet (AA-) for indicated times. n=3 completely independent experiments on independent samples. Significance values are calculated by a two -tailed student's t-test assuming unequal variance. Error bars represent one standard deviation.

F: Percentage of proteasome activity in cytosolic (-mitochondria) and mitochondrial fractions from mixed egg samples. n=3 completely independent experiments on independent samples

G: Assays using UbiQ-018 activity probe which detects all three active sites of the proteosome measuring proteasome activity in active and quiescent oocytes.

H: Quantification of the three activities of the proteasome (trypsin, chymotrypsin, and caspase) for active and quiescent oocytes (n=6 completely independent experiments on independent samples).

Supplementary Figure 2

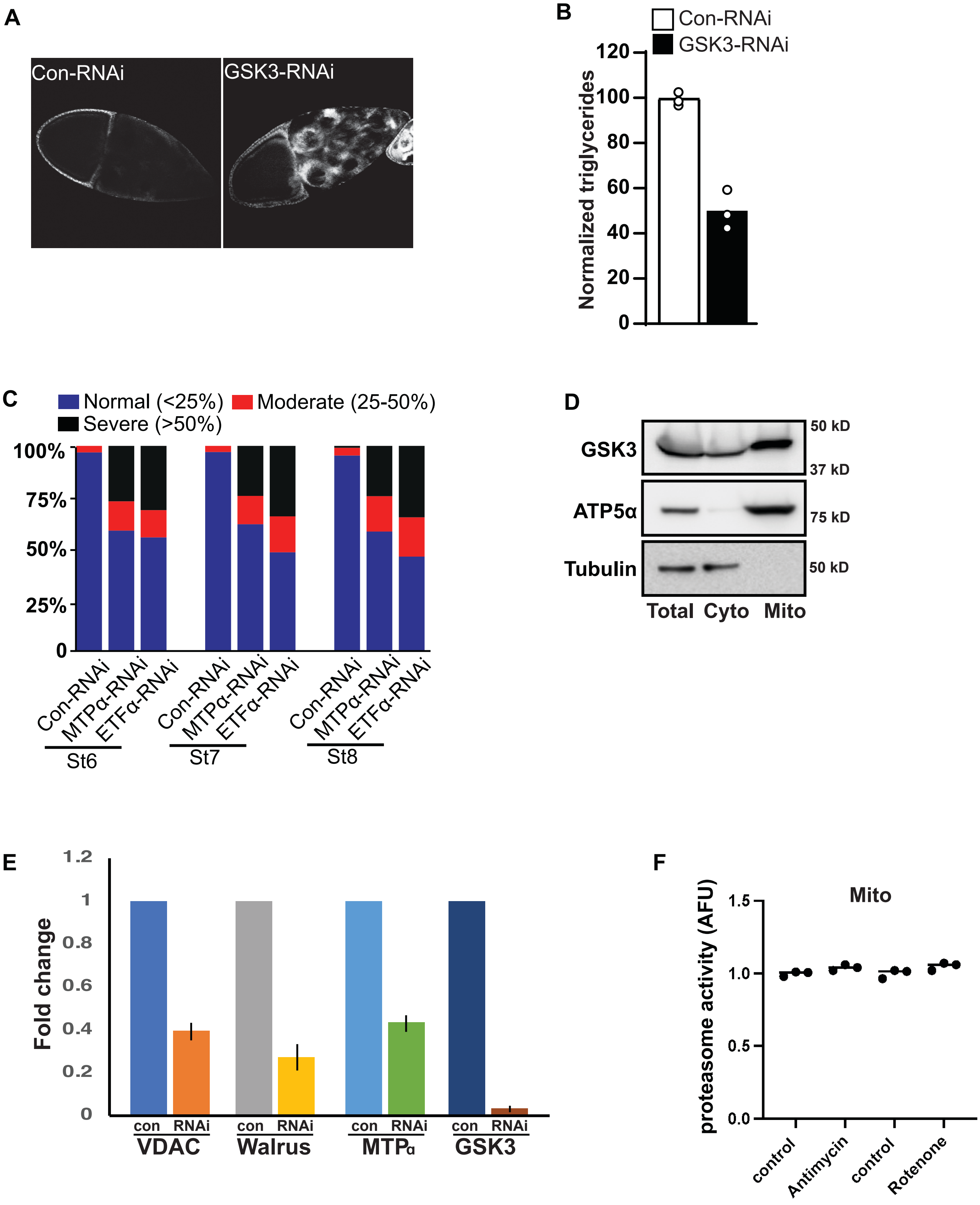

Supplementary Figure 2

A: TMRE staining of a control-RNAi and GSK3-RNAi ovarioles.

B: Normalized triglyceride levels in st14 eggs from a control-RNAi and GSK3-RNAi ovarioles (n=8 independent samples).

C: Quantification of TMRE staining from staged MTPa-RNAi and ETFa-RNAi egg chambers(n>40 ovarioles).

D: Western blotting to detect GSK3 protein levels in total, cytosolic and mitochondrial fractions from wild-type ovarioles(n=3 independent experiments).

E: Summary of effects of RNAi knockdown of either MTPα or ETFα on mRNA levels (n=3 independent experiments).

F: Mitochondrial associate proteasome activity from cells treated with either 0.3uM Rotenone or 0.3uM Antimycin A for 6 hrs(n=3 independent experiments). Significance values are calculated by a two -tailed student's t-test assuming unequal variance.

Supplementary Figure 3

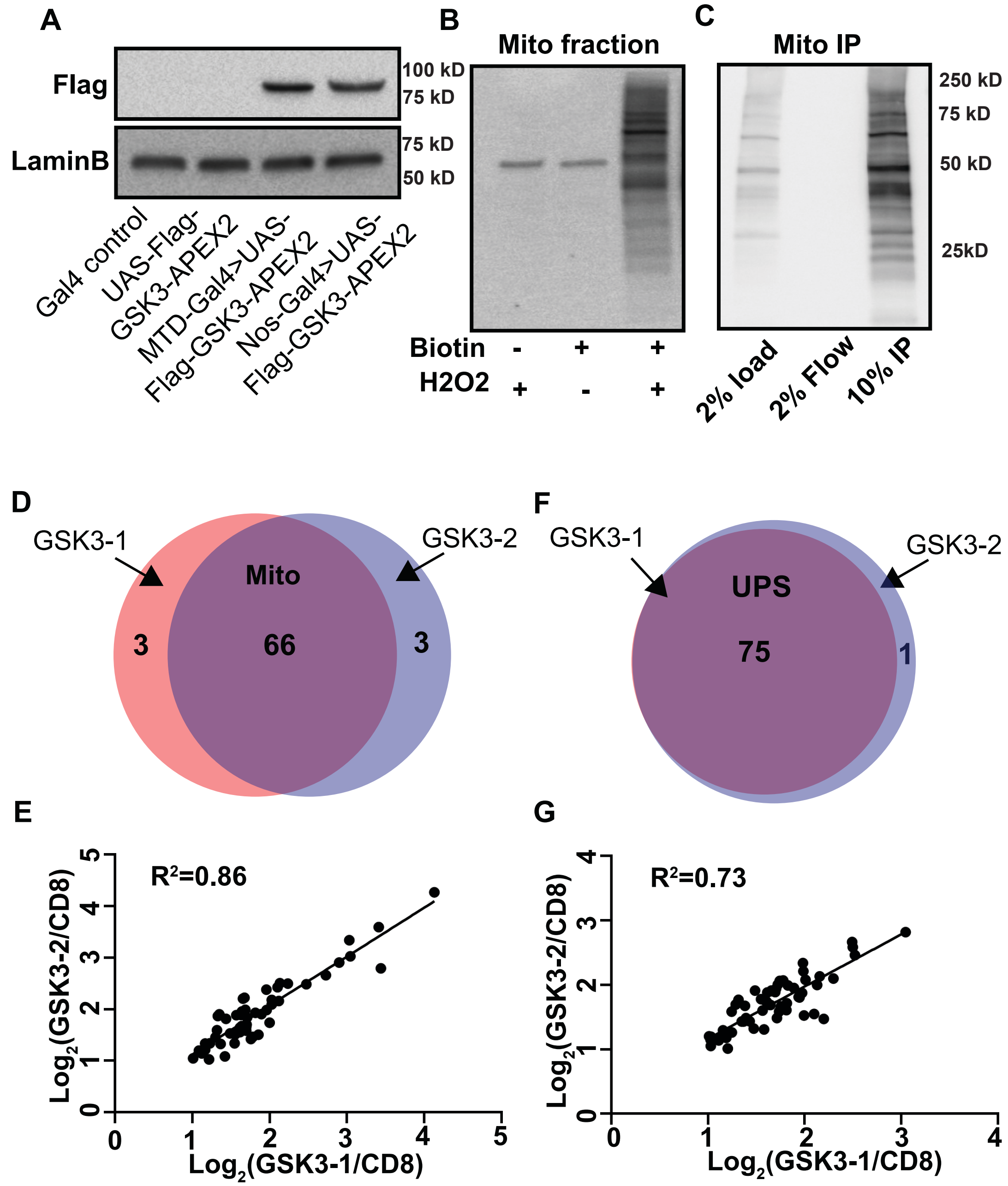

Supplementary Figure 3

A-C: Validation of GSK3-APEX2 expression, biotinylation labeling specificity, and pulldown efficiency.

D and E: Overlap (D) and correlation analysis (E) for two biological replicates of mitochondria-associated proteins identified by GSK3-Apex2.

F and G: Overlap (F) and correlation analysis (G) for two biological replicates of UPS system proteins identified by GSK3-Apex2.

Supplementary Figure 4

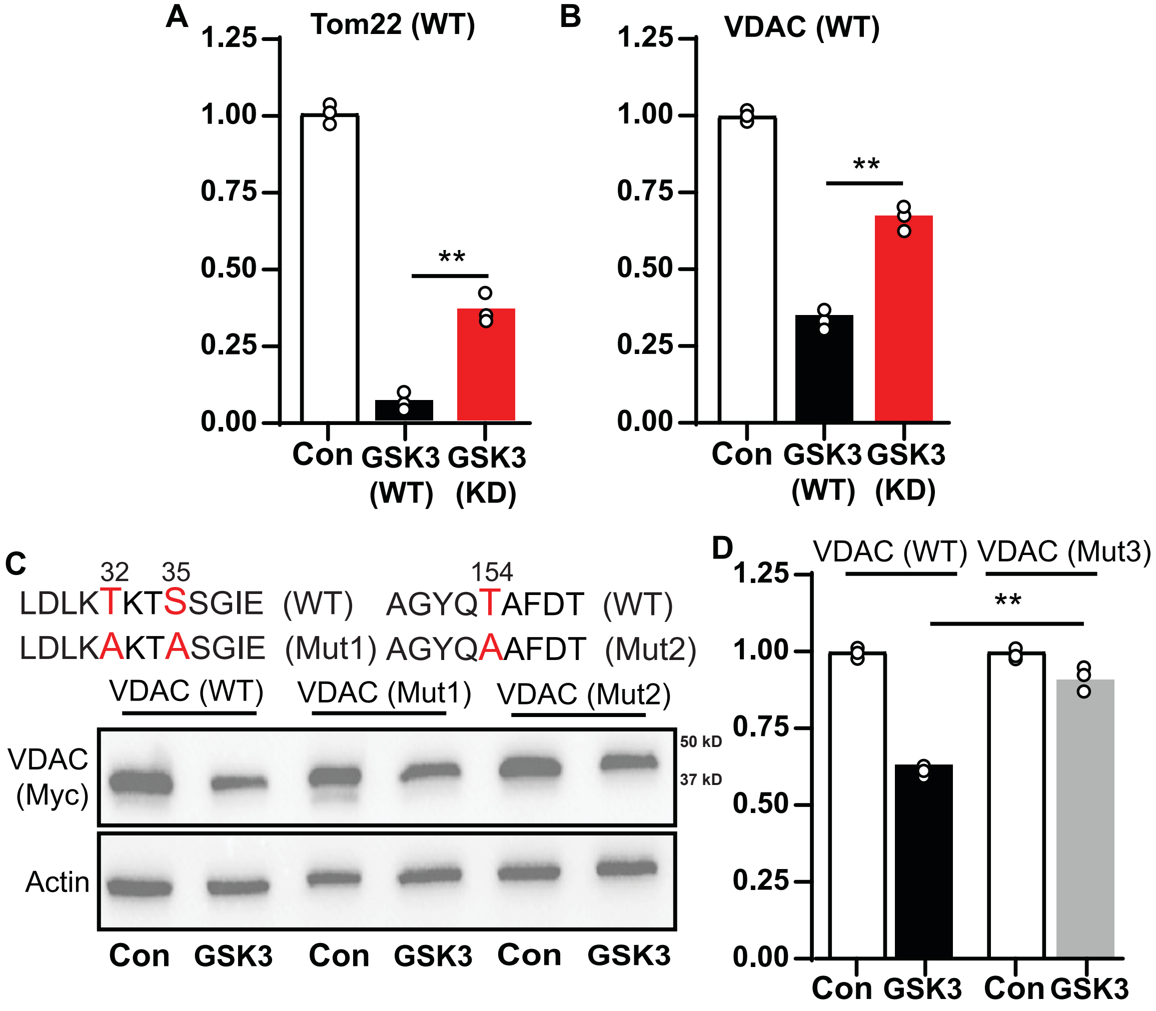

Supplementary Figure 4

A and B: Quantification of TOM22 (A) and VDAC (B) protein levels in control 293T cells or cells expressing wild-type GSK3 (WT) or kinase-dead GSK3 (KD). n=3 completely independent experiments.

C: Western blotting to detect wild-type VDAC (WT) and indicated phosphorylation site mutant VDACs (S/T->A) in control 293T cells and cells expressing wild-type GSK3 (WT) (n=3 independent experiments).

D: Quantification of wild-type VDAC (WT) and indicated phosphorylation site mutant VDAC (mut3) protein levels in control 293T cells and cells expressing wild-type GSK3 (WT). n=3 completely independent experiments. Significance values are calculated by a two -tailed student's t-test assuming unequal variance.

Supplementary Figure 5

A

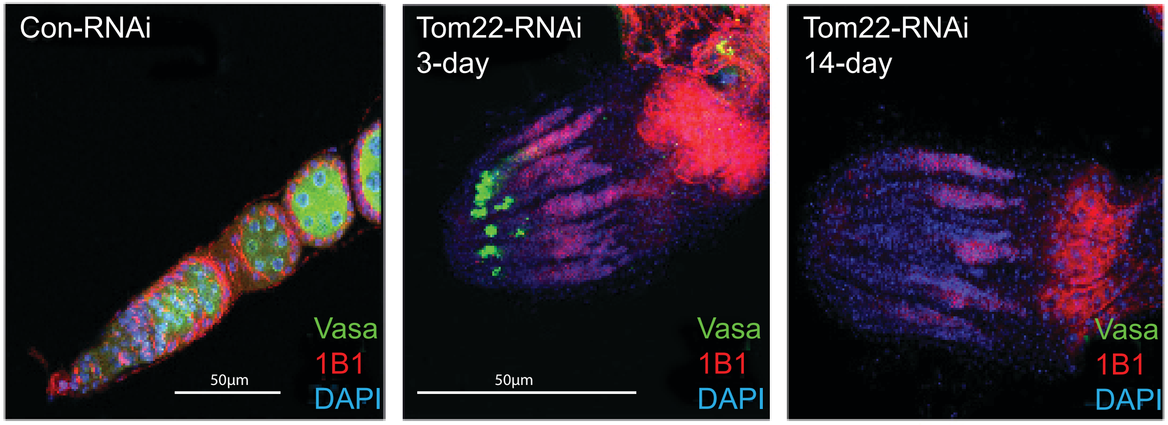

B

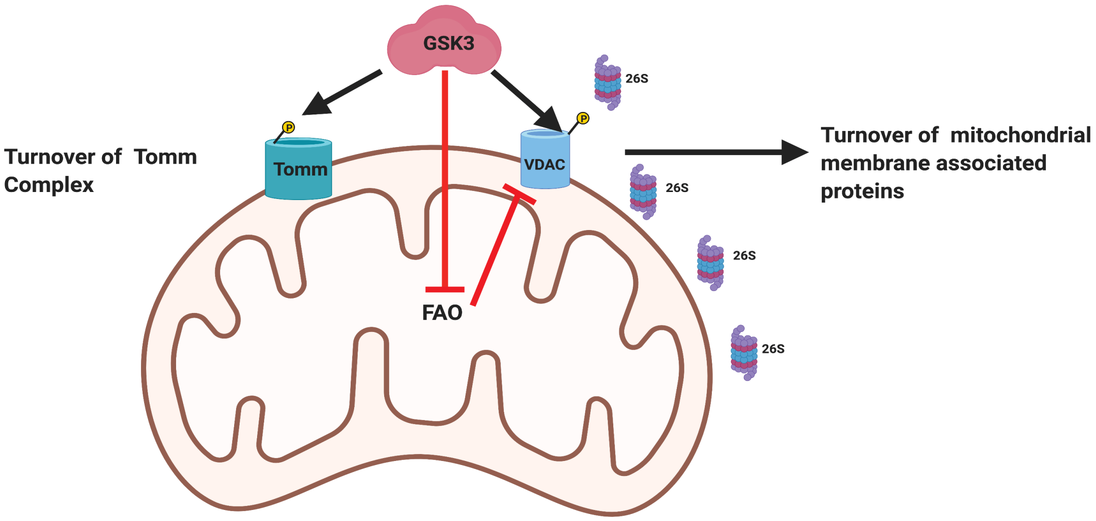

C

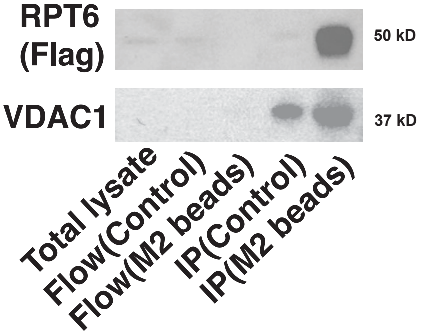

Supplementary Figure 5

A: Immunostaining to detect VASA (green), *hts* (red), and DAPI (blue) in control-RNAi and Tom22-RNAi ovarioles at 3 and 14 days.

B: A model for GSK3 regulation of mitochondrial remodeling during quiescence. Created with BioRender.com

C: CO-IP assay using a RPT6-flag cell line to determine if VDAC1 associates with the proteasome in quiescent cells(n=3 independent experiments).

Supplementary Figure 6

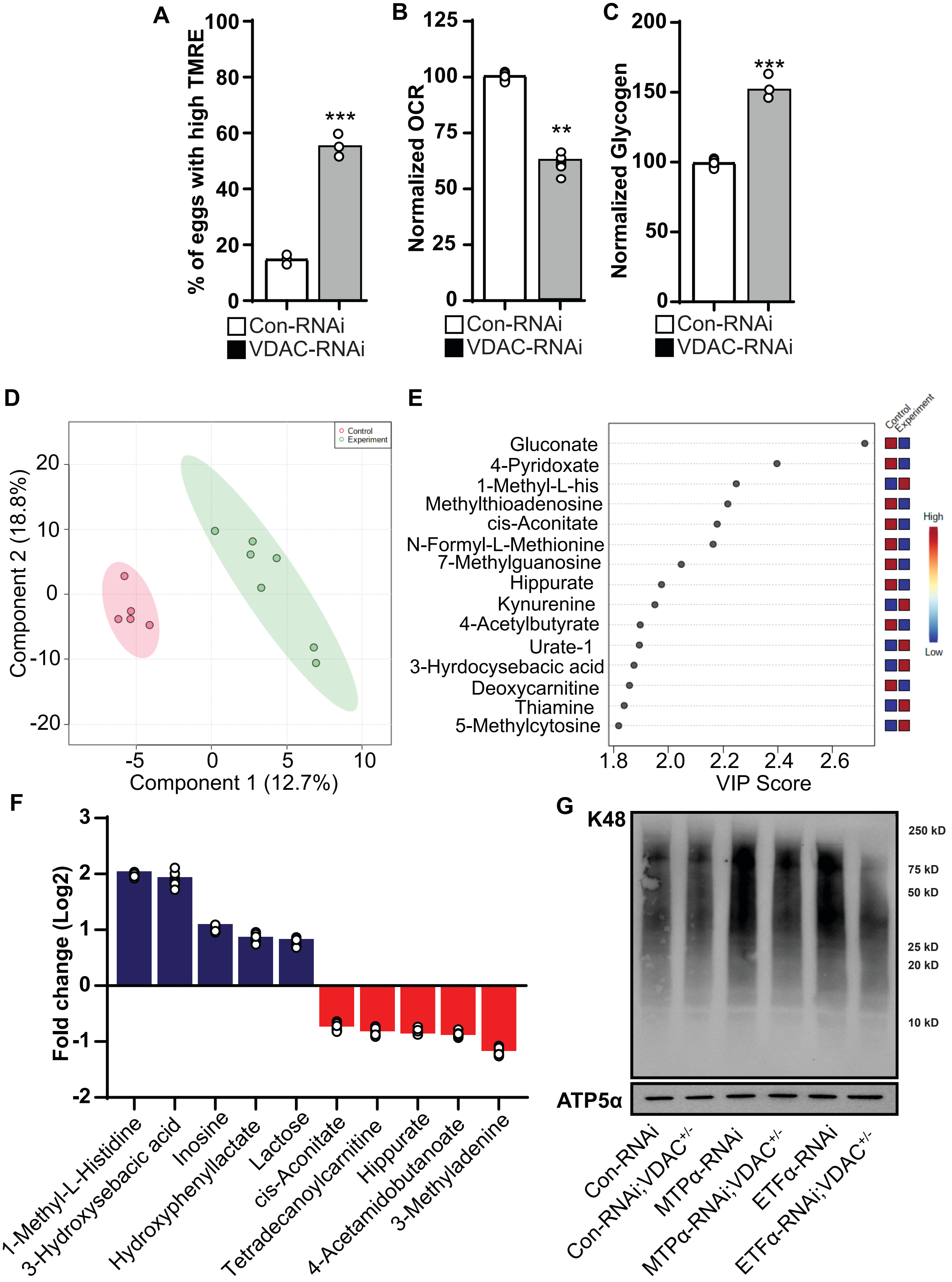

Supplementary Figure 6

A and B: Effects of VDAC-RNAi on TMRE mitochondrial membrane potential staining (3 experiments of N=>40 egg chambers) (A) and OCR (N=4 experiments conducted on independent biological replicates) (B) in mitochondria from st10 eggs.

C: Normalized glycogen levels in st14 eggs from control-RNAi and VDAC-RNAi ovarioles n=3 independent experiments .

D-F: Summary of metabolic analysis of st14 eggs from control-RNAi and VDAC-RNAi ovarioles.

G: Measurements of K48 ubiquitin levels in epistasis analysis of VDAC and FAO pathway in regulating mitochondria-associated proteasome activity(n=3 independent experiments). Significance values are calculated by a two -tailed student's t-test assuming unequal variance.

Supplementary Figure 7

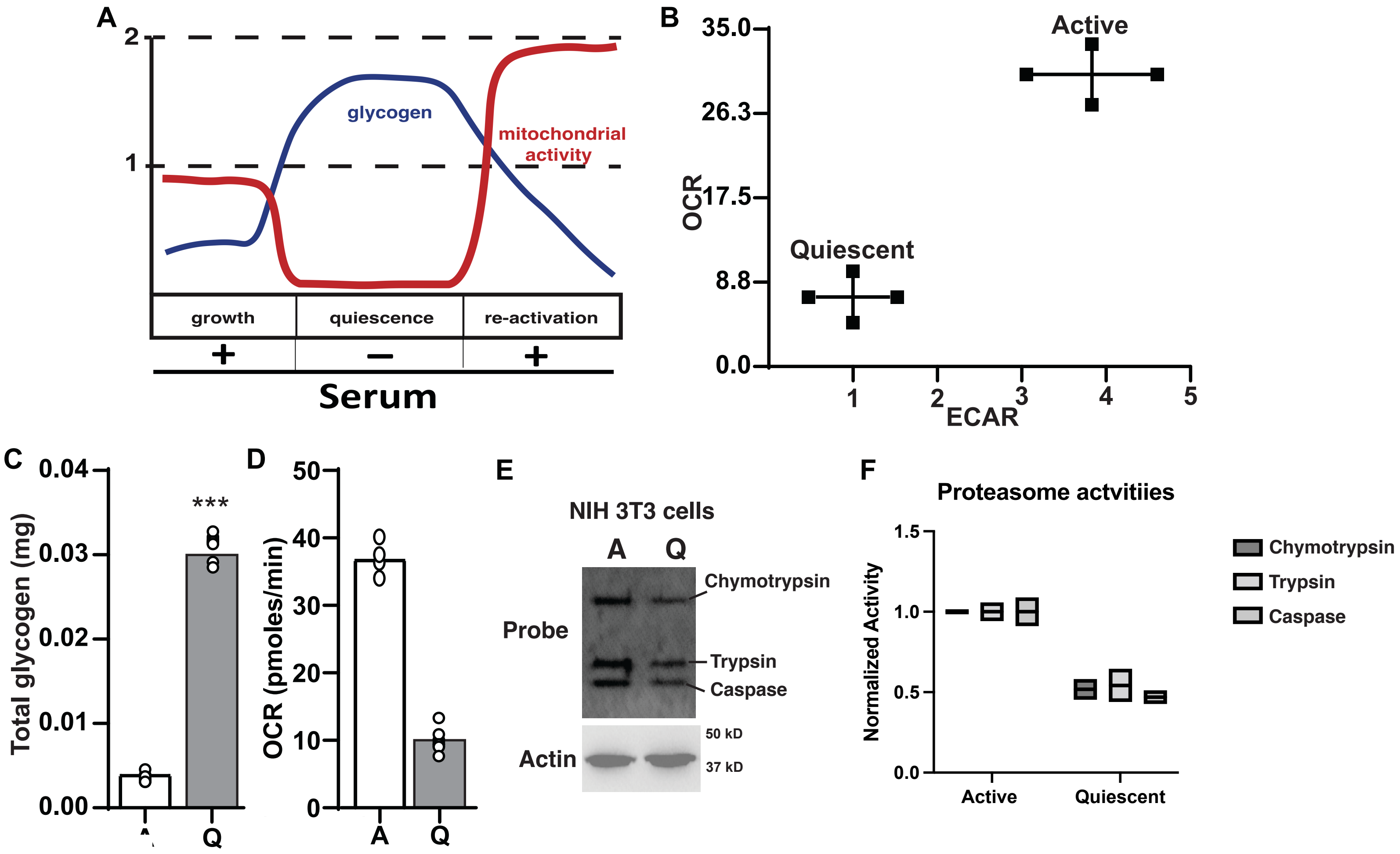

G

Term	Count	Pvalue	Fold enrichment	FDR
Mitochondrion	93	7.00E-125	21.4	9.20E-123
Transit peptide	57	5.40E-69	27.3	3.60E-67
Acetylation	75	4.10E-47	5.9	1.80E-45
mitochondrial inner membrane	42	1.30E-45	22.9	7.50E-44
mitochondrial matrix	25	3.40E-28	28.1	1.30E-26
Mitochondrion inner membrane	26	5.60E-28	25	1.90E-26
myelin sheath	24	2.30E-26	26.4	6.80E-25
Metabolic pathways	43	5.40E-19	4.1	3.30E-17
Carbon metabolism	19	6.00E-19	19.7	3.30E-17
tricarboxylic acid cycle	12	1.00E-18	80.5	5.50E-16
oxidation-reduction process	29	1.90E-18	8.3	5.50E-16
Tricarboxylic acid cycle	11	2.30E-18	107.3	6.00E-17
Oxidoreductase	25	5.10E-17	9.5	1.10E-15
metabolic process	24	7.30E-17	10.1	1.40E-14
mitochondrial outer membrane	16	4.40E-16	22.1	1.00E-14
FAD	14	1.60E-15	28.9	3.00E-14
Biosynthesis of antibiotics	20	2.60E-15	11.2	7.10E-14
Phosphoprotein	69	2.70E-15	2.2	4.50E-14
oxidoreductase activity	25	2.90E-15	7.9	7.00E-13
Flavoprotein	14	6.00E-15	26.3	8.80E-14

Supplementary Figure 7

A: A model depicting the changes in mitochondrial activity and glycogen storage observed as cells transition from growth to quiescence and from quiescence to the reactivation of growth.

B: An energy map examining OCR and ECAR for actively growing and quiescent NIH 3T3 cells.

C and D: Glycogen (C) (n= 3 independent experiments) and OCR (D) measurements for actively growing and quiescent NIH 3T3 cells.

E: Assays using UbiQ-018 activity probe (which detects all three active sites of the proteosome) measuring proteasome activity in active and quiescent oocytes(n=2 independent experiments).

F: Quantification of the three activities of the proteasome (trypsin, chymotrypsin, and caspase) for active and quiescent oocytes(n=2 completely independent experiments on independent samples)..

G: GO term analysis of mitochondrial proteins identified in quiescent NIH 3T3 cells compared with active 3T3 cells. Significance values are calculated by a two -tailed student's t-test assuming unequal variance.
