# Supplementary material for: Highly conserved shifts in ubiquitin-proteasome system (UPS) activity drive mitochondrial remodeling during quiescence
Source: Nat Commun. 2022 Aug 1;13:4462. doi: 10.1038/s41467-022-32206-2 (PMC9343427; doi:10.1038/s41467-022-32206-2)
Supplement: Supplementary file 3 — Description of Additional Supplementary Files [file 41467_2022_32206_MOESM3_ESM.pdf]

Description of Additional Supplementary Files

File name: Supplementary Data 1

Description: RNA sequencing data from Sieber and Spradling 2015 examining gene expression of UPS components in stage 8 egg chambers and stage 10 oocytes that are beginning to enter quiescence. Stage 10 was chosen to capture the transcriptional profile at the first point where MRQ begins and oocyte transcription is suppressed. Significance was determined FDR adjusted Pvalue to account for multiple sampling. All genes in this table display a FDR of less than .05.

File name: Supplementary Data 2

Description: A list of mitochondrial proteins that associate with GSK3 in mitochondrial fractions based on proximity labeling. The list displayed represents the list of proteins that show greater than 2-fold increase in the GSK3-APEX/CD8-APEX ratio in 2 independent experiments.

File name: Supplementary Data 3

Description: A list of ubiquitin proteasome system proteins that associate with GSK3 in mitochondrial fractions based on proximity labeling. The list displayed represents the list of proteins that show greater than 2-fold increase in the GSK3-APEX/CD8-APEX ratio in 2 independent experiments.

File name: Supplementary Data 4

Description: A list of mitochondrial downregulated in mitochondria isolated from quiescent 3T3 cells as assayed by mitochondrial proteomics. n=3 completely independent experiments. Significance was determined by FDR-adjusted Pvalue.

File name: Supplementary Data 5

Description: A list of oligos and cDNAs used in this study.
