# Supplementary material for: Highly conserved shifts in ubiquitin-proteasome system (UPS) activity drive mitochondrial remodeling during quiescence
Source: Nat Commun. 2022 Aug 1;13:4462. doi: 10.1038/s41467-022-32206-2 (PMC9343427; doi:10.1038/s41467-022-32206-2)
Supplement: Supplementary file 9 — Reporting Summary [file 41467_2022_32206_MOESM9_ESM.pdf]

nature portfolio

Corresponding author(s): Matthew Sieber

Last updated by author(s): May 3, 2022

Reporting Summary

Nature Portfolio wishes to improve the reproducibility of the work that we publish. This form provides structure for consistency and transparency in reporting. For further information on Nature Portfolio policies, see our Editorial Policies and the Editorial Policy Checklist.

Statistics

For all statistical analyses, confirm that the following items are present in the figure legend, table legend, main text, or Methods section.

n/a	Confirmed	
☐	☒	The exact sample size (*n*) for each experimental group/condition, given as a discrete number and unit of measurement
☐	☒	A statement on whether measurements were taken from distinct samples or whether the same sample was measured repeatedly
☐	☒	The statistical test(s) used AND whether they are one- or two-sided<br>*Only common tests should be described solely by name; describe more complex techniques in the Methods section.*
☐	☒	A description of all covariates tested
☐	☒	A description of any assumptions or corrections, such as tests of normality and adjustment for multiple comparisons
☐	☒	A full description of the statistical parameters including central tendency (e.g. means) or other basic estimates (e.g. regression coefficient) AND variation (e.g. standard deviation) or associated estimates of uncertainty (e.g. confidence intervals)
☐	☒	For null hypothesis testing, the test statistic (e.g. F, t, r) with confidence intervals, effect sizes, degrees of freedom and P value noted<br>*Give P values as exact values whenever suitable.*
☒	☐	For Bayesian analysis, information on the choice of priors and Markov chain Monte Carlo settings
☒	☐	For hierarchical and complex designs, identification of the appropriate level for tests and full reporting of outcomes
☐	☒	Estimates of effect sizes (e.g. Cohen's *d*, Pearson's *r*), indicating how they were calculated

Our web collection on statistics for biologists contains articles on many of the points above.

Software and code

Policy information about availability of computer code

Data collection	Sciex SIMCA, MassLynx 4.1, QuanLynx,
Data analysis	Prism, Excel,

For manuscripts utilizing custom algorithms or software that are central to the research but not yet described in published literature, software must be made available to editors and reviewers. We strongly encourage code deposition in a community repository (e.g. GitHub). See the Nature Portfolio guidelines for submitting code & software for further information.

Data

Policy information about availability of data

All manuscripts must include a data availability statement. This statement should provide the following information, where applicable:

- Accession codes, unique identifiers, or web links for publicly available datasets
- A description of any restrictions on data availability
- For clinical datasets or third party data, please ensure that the statement adheres to our policy

Our proteomic data sets are available on line at massive.ucsd.edu under the accession numbers MSV000087642 and MSV000087641. All raw data is provided in source data.

Field-specific reporting

Please select the one below that is the best fit for your research. If you are not sure, read the appropriate sections before making your selection.

☒ Life sciences ☐ Behavioural & social sciences ☐ Ecological, evolutionary & environmental sciences

For a reference copy of the document with all sections, see nature.com/documents/nr-reporting-summary-flat.pdf

Life sciences study design

All studies must disclose on these points even when the disclosure is negative.

Sample size	Sample size was dictated by the standards in the filed and our ability to isolate quiescent cells. Wojcik, C., DeMartino, G.N. (2002)J. Biol. Chem. 277(8): 6188--6197. and Sieber et al (2016) Cell 164(3): 420--432.
Data exclusions	No data was excluded from this study
Replication	No replicate experiments were excluded from this study. All replicate experiments displayed effects similar to those presented in the manscript.
Randomization	Our proteomics samples were randomized and blinded to ensure no bias in subsequent analysis
Blinding	Our proteomics samples were randomized and blinded to ensure no bias in subsequent analysis

Reporting for specific materials, systems and methods

We require information from authors about some types of materials, experimental systems and methods used in many studies. Here, indicate whether each material, system or method listed is relevant to your study. If you are not sure if a list item applies to your research, read the appropriate section before selecting a response.

Materials & experimental systems

n/a	Involved in the study
☐	☒ Antibodies
☐	☒ Eukaryotic cell lines
☒	☐ Palaeontology and archaeology
☐	☒ Animals and other organisms
☒	☐ Human research participants
☒	☐ Clinical data
☒	☐ Dual use research of concern

Methods

n/a	Involved in the study
☒	☐ ChIP-seq
☒	☐ Flow cytometry
☒	☐ MRI-based neuroimaging

Antibodies

Antibodies used	Anti-ATP5a (Abcam #15H4C4 lot#GR3306993-25), Anti-K48 linkage (ABcam #EP8589 lot:6),anti-20S (George Demartino), anti-Rpt2(George Demartino), anti-Rpt5 (George Demartino), anti-Rpn12 (George Demartino), anti-tubulin (DSHB# E7), Anti-actin(DSHB# JLA20)
Validation	These antibodies were validated in multiple cells lines and drosophila by Western blot. Cells were also confirm free of mycoplasma by commercial kit.

Eukaryotic cell lines

Policy information about cell lines

Cell line source(s)	3t3 cells (ATCC CRL-1658), HEK293T cells (ATCC CRL-3216)
Authentication	These cells were validated by their ability to differentiate into myofibers in vitro.
Mycoplasma contamination	No contamination was detected
Commonly misidentified lines (See ICLAC register)	no commoly misidentified lines were used in this study

Animals and other organisms

Policy information about studies involving animals; ARRIVE guidelines recommended for reporting animal research

Laboratory animals	drosophila (oregon R) adult females, neurospora crassa
Wild animals	N/A
Field-collected samples	N/A
Ethics oversight	No ethical guidance was required for this study.

Note that full information on the approval of the study protocol must also be provided in the manuscript.
